# Anti-Inflammatory Effect of Dimethyl Fumarate Associates with the Inhibition of Thioredoxin Reductase 1 in RAW 264.7 Cells

**DOI:** 10.3390/molecules28010107

**Published:** 2022-12-23

**Authors:** Rui Yang, Shibo Sun, Yining Guo, Yao Meng, Haowen Liu, Meiyun Shi, Shui Guan, Jianqiang Xu

**Affiliations:** 1 School of Life and Pharmaceutical Sciences (LPS), Panjin Institute of Industrial Technology (PIIT), Liaoning Key Laboratory of Chemical Additive Synthesis and Separation, Dalian University of Technology, Panjin 124221, China; 2 State Key Laboratory of Fine Chemicals, Dalian R&D Center for Stem Cell and Tissue Engineering, School of Chemical Engineering, Dalian University of Technology, Dalian 116023, China; 3 Research & Educational Center for the Control Engineering of Translational Precision Medicine, School of Biomedical Engineering, Dalian University of Technology, Dalian 116024, China

**Keywords:** dimethyl fumarate, thioredoxin reductase (TXNRD), immunometabolite, selenoprotein, anti-inflammation, redox regulation

## Abstract

Macrophages secrete a variety of pro-inflammatory cytokines in response to pathogen-associated molecular patterns (PAMPs) and damage-associated molecular patterns (DAMPs) but abnormal release of cytokines unfortunately promotes cytokine storms. Dimethyl fumarate (DMF), an FDA-approved drug for multiple sclerosis (MS) treatment, has been found as an effective therapeutic agent for resolution. In this study, the anti-inflammatory effect of DMF was found to correlate to selenoprotein thioredoxin reductase 1 (TXNRD1). DMF irreversibly modified the Sec^498^ residue and C-terminal catalytic cysteine residues of TXNRD1 in a time- and dose-dependent manner. In LPS-stimulated RAW 264.7 cells, cellular TXNRD activity was increased through up-regulation of the protein level and DMF inhibited TXNRD activity and the nitric oxide (NO) production of RAW 264.7 cells. Meanwhile, the inhibition of TXNRD1 by DMF would contribute to the redox regulation of inflammation and promote the nuclear factor erythroid 2-related factor 2 (NRF2) activation. Notably, inhibition of cellular TXNRD1 by auranofin or TRi-1 showed anti-inflammatory effect in RAW 264.7 cells. This finding demonstrated that targeting TXNRD1 is a potential mechanism of using immunometabolites for dousing inflammation in response to pathogens and highlights the potential of TXNRD1 inhibitors in immune regulation.

## 1. Introduction

Selenoprotein thioredoxin reductase 1 (TXNRD1, encoded by *txnrd1*) is a basal antioxidant enzyme that participates in multiple cellular processes, such as antioxidant defense, programmed cell death, and regulation of cell fate [1,2]. TXNRD1 can scavenge excessive reactive oxygen species (ROS) by reducing and recycling thioredoxin (TXN, encoded by *txn*) or 14 kDa thioredoxin-related protein (TRP14, encoded by *txndc17*), or directly quenching free radicals through reacting with compounds or biomolecules [3,4,5]. Since the TXN system was known to modulate gene expression by promoting the activity of transcription factors such as NF-κB and HIF-1α, TXNRD1 was heretofore considered as a negative regulator of KEAP1-NRF2-ARE antioxidant pathway [6,7].

Macrophages (Mφ) and dendritic cells (DCs) secrete several cytokines in response to pathogen-associated molecular patterns (PAMPs) and damage-associated molecular patterns (DAMPs) by stimulating Toll-like receptors (TLRs), which activate NF-κB-dependent transcription of pro-inflammatory cytokines including IL-6, TNF-a and IL-1β [8,9]. TLRs stimulation increases the generation of ROS and redox-related proteins, such as TXN, TXNRD1 and peroxiredoxins (Prxs), which regulate inflammation and resolution by altering nucleotide synthesis and NADPH pools, or the rewired TCA cycle and the production of functional immunometabolites, such as itaconate and succinate [10,11,12,13,14,15,16].

Dimethyl fumarate (DMF), as a derivative of fumarate, has been used as an efficient chemotherapy for multiple sclerosis (MS) [17]. DMF possesses the capacity to bind NF-κB and inhibits GAPDH, suppressing pro-inflammatory cytokines production and aerobic glycolysis flux [18,19,20]. Meanwhile, DMF activates NRF2 to produce antioxidant and anti-inflammatory effects. Although the activation of NRF2 by DMF is mainly caused by the succinylation of KEAP1, DMF also binds to TXN1, a negative regulator of the KEAP1-NRF2-ARE antioxidant system, which contributes to its action [21,22]. Recently, the TXN system was reported to be related to the immunogenic cell death of cancer cells, suggesting promising roles of TXN and TXNRD1 for immune modulation [23,24,25]. However, as regards the center enzyme in the TXN system, the interaction between TXNRD1 and DMF has not been fully revealed.

In this study, we investigated the effects of DMF on RAW 264.7 cells and found that DMF exhibits an inhibitory effect on TXNRD1. Our results showed that DMF irreversibly modified the Sec^498^ residue and C-terminal catalytic Cys residues of TXNRD1 which contribute to NRF2 activation and the anti-inflammation effects of DMF. The present findings may uncover the targeting of DMF on TXNRD1 and point out a potential mechanism of immunometabolite derivatives in response to pathogens.

## 2. Results

### 2.1. DMF Is Not a Substrate but an Inhibitor of TXNRD1

**Figure 1:**
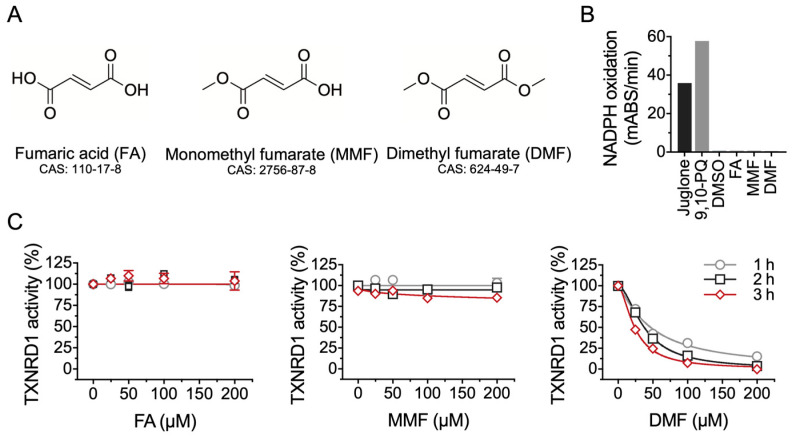
DMF is an inhibitor of TXNRD1. (**A**) Chemical structures of FA, MMF and DMF. (**B**) NADPH oxidation activity of TXNRD1 using various compounds as electron acceptors. 30 μM juglone and 30 μM 9,10-PQ were used as positive controls in this experiment. FA, MMF and DMF were included for testing. The final mixture solution contained 30 nM TXNRD1, 200 μM NADPH and 200 μM compounds. (**C**) Dose- and time-dependent inhibition of DMF on TXNRD1. Rat TXNRD1 was incubated with FA, MMF and DMF for the indicated time. The TXNRD1 activity was assayed by classic DTNB reducing activity assay. Data are presented as mean ± SEM with three independent experiments.

Monomethyl fumarate (MMF) and DMF are derivatives of metabolite fumaric acid (FA), as shown in Figure 1A. Regarding the possible interactions between TXNRD1 and DMF, we first questioned whether the three compounds are substrates of TXNRD1, able to accept electrons from NADPH. Two canonical TXNRD1 substrates, 9,10-PQ and juglone, were used in this experiment and NADPH consumption was observed using recombinant rat TXNRD1. However, FA or MMF or DMF failed to display any NADPH consumption, indicating that FA and its derivatives MMF and DMF were not substrates of the enzyme (Figure 1B). Then we tested the inhibitory effect of these three molecules on recombinant rat TXNRD1. After incubation for the indicated time, DMF but not MMF and FA, significantly inhibited the TXNRD1 based on the DTNB reducing activity assay; the IC_50_ value was approx. 30 μM, depending on the duration of incubation (Figure 1C). This result showed that DMF is an inhibitor of TXNRD1 and not involved in the redox recycling of the enzyme.

### 2.2. DMF Irreversibly Inhibits TXNRD1 Activity but Shows Much Less Inhibition on GSR

**Figure 2:**
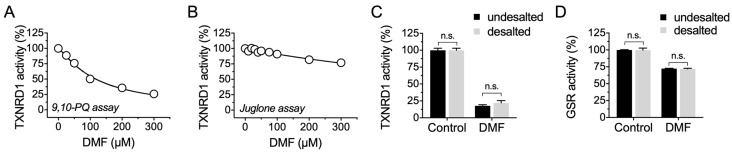
DMF irreversibly inhibits TXNRD1 activity but shows less inhibition on GSR. (**A**,**B**) Inhibition of 9,10-PQ and juglone reducing activity of TXNRD1 by DMF. Rat TXNRD1 (0.3 μM) was treated with DMF for 1 h, and then the 9,10-PQ and juglone reducing activities were assayed, respectively. (**C**) Irreversible inhibition of DMF on TXNRD1. TXNRD1 was treated with 100 μM DMF for 2 h and then desalted using NAP^TM^-5 desalting column. The residual TXNRD1 activity was measured by DTNB reducing activity. (**D**) Less inhibition of DMF on GSR. Yeast GSR was incubated with 500 μM DMF for 2 h and then desalted using NAP^TM^-5 desalting column. The activity of GSR was determined in presence of 200 μM NADPH by reducing GSSG. Data are presented as mean ± SEM with three independent experiments. n.s., not significant.

We then investigated the inhibition mechanism of DMF on TNXRD1. In addition to the DTNB assay, the 9,10-PQ reducing activity, a highly Sec-dependent substrate of TXNRD1, was also inhibited by DMF (Figure 2A), whereas the juglone reduction activity of TXNRD1 was not so greatly inhibited (Figure 2B). After removing the dissociative DMF from the incubation system using a NAP^TM^-5 desalting column, the residual activity was not significantly rescued, indicating an irreversible inhibition of DMF on TXNRD1 (Figure 2C). Glutathione reductase (GSR, encoded by *gsr*) is a flavoprotein akin to TXNRD1, but lacking selenocysteine at its C-terminus. Using a high concentration (up to 500 μM), DMF showed irreversible inhibition towards the GSSG reducing activity of GSR (Figure 2D).

### 2.3. DMF Modifies the Selenocysteine and Cysteine Residues of TXNRD1

**Figure 3:**
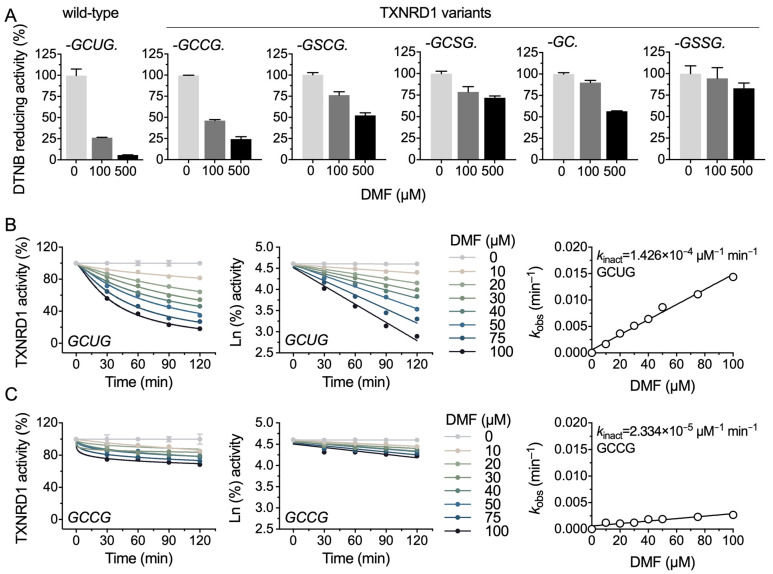
The C-terminal redox motif of TXNRD1 is more prone to be targeted by DMF than N-terminal domains. (**A**) Inhibition of TXNRD1 variants by DMF. Wild-type TXNRD1 (0.1 μM) and its mutant variants (1 μM) were pre-reduced by 100 μM NADPH, and then treated with DMF for 2 h; the remained activity was determined by DTNB reducing activity. The final reaction mixture contained 10 nM wild-type TXNRD1 or 100 nM TXNRD1 mutant variants. (**B**,**C**) Time-dependent inhibition of DMF on wild-type rat TXNRD1 and its GCCG mutant. 0.2 μM wild-type TXNRD1 and 2 μM its GCCG mutant were pre-reduced by NADPH and then treated with DMF for the indicated time. The residual enzyme activity was assayed by DTNB reducing assay. Data are presented as mean ± SEM with three independent experiments.

TXNRD1 contains a N-terminal GSR-like “-CVNVGC-” motif and a C-terminal Sec-containing “-GCUG” redox motif. To reveal the DMF inhibition in detail and identify the key residues of DMF targeting TXNRD1, we prepared five mutant variants of rat TXNRD1 and further analyzed the inhibition. Compared to the wild-type enzyme (-GCUG), the Sec-deficient mutation of TXNRD1 (-GCCG, -GSCG, -GCSG, -GC and -GSSG) conferred resistance to DMF, suggesting DMF mainly targets the Sec^498^ residue of TXNRD1 (Figure 3A). However, the Sec-deficient TXNRD1 mutant variants were inhibited by DMF, indicating that DMF can modify the C-terminal cysteine residues or even the N-terminal cysteine residues of TXNRD1 (Figure 3A), which is consistent with the result that DMF inhibited GSR activity. These results elucidated that DMF inhibits both the C-terminal reducing activity and N-terminal NADPH oxidase activity of TXNRD1.

To reveal the inhibitory details of DMF on TXNRD1, we determined the *k*_inact_ of DMF on wild-type TXNRD1 and its GCCG mutant (Sec^498^ to Cys^498^, U498C). The *k*_inact_ of the two variants was 1.426 × 10^−4^ and 2.334 × 10^−5^ μM^−1^ min^−1^, respectively. The six-fold difference in *k*_inact_ indicated the preferentially targeted residue of TXNRD1 by DMF (Figure 3B,C).

### 2.4. DMF Inhibits Three Species of TXNRD1

**Figure 4:**
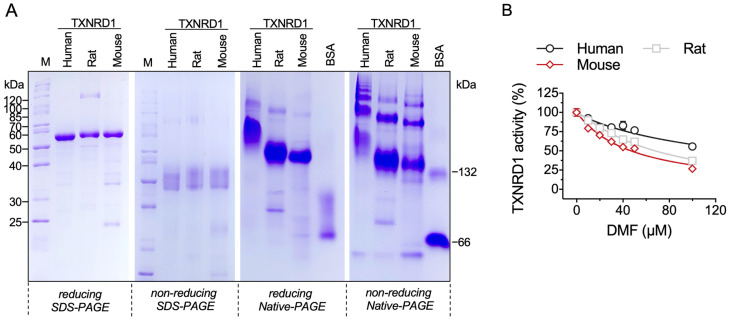
Inhibition of human TXNRD1 by DMF. (**A**) SDS-PAGE and Native-PAGE analysis of human, rat and mouse TXNRD1. (**B**) Inhibition of three species of TXNRD1 by DMF. The final reaction mix contained 40 nM human TXNRD1, 10 nM rat TXNRD1 and 20 nM mouse TXNRD1, respectively.

Rat TXNRD1 is highly homologous to human and mouse species, regarding the amino acid sequence. To validate the inhibitory effect and targeting priority of DMF, we recombinantly expressed and purified human TXNRD1 and mouse TXNRD1 and performed the SDS-PAGE analysis and Native-PAGE analysis together with rat TXNRD1 (Figure 4A). In parallel, we tested the inhibition of DMF on TXNRD1 from three species. DMF showed inhibition towards the three TXNRD1s. The specific activity of the enzymes was 8 U/mg for human TXNRD1, 11 U/mg for mouse TXNRD1 and 22.7 U/mg for rat TXNRD1 (Figure 4B).

### 2.5. LPS Stimulation Increases Cellular TXNRD1 Activity through Up-Regulated TXNRD1 Level

**Figure 5:**
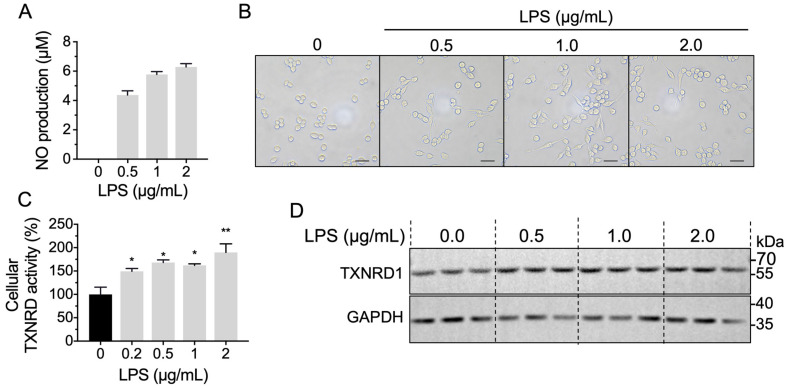
LPS-stimulation increases cellular TXNRD activity. (**A**) NO production of LPS-simulated RAW 264.7 cells. RAW 264.7 cells were treated with LPS for 24 h and the NO production was assayed. (**B**) Cellular morphology image of RAW 264.7 cells upon LPS treatment for 6 h. Scale bar represents 200 μm. (**C**,**D**) Cellular TXNRD activity and TXNRD1 protein level were determined upon LPS treatment for 6 h in RAW 264.7 cells. *, *p* < 0.05, **, *p* < 0.01.

We were curious whether the anti-inflammation effect of DMF is correlated with the inhibition on TXNRD1. Upon LPS treatment, the cellular morphology of RAW 264.7 cells was changed from round morphology into spindly morphology and the NO production was significantly increased, indicating the polarization of macrophages into M1 type (Figure 5A,B). Meanwhile, the cellular TXNRD1 activity was significantly increased, and the protein level of TXNRD1 was up-regulated (Figure 5C,D).

### 2.6. Anti-Inflammation Effect of DMF Is Correlated with TXNRD1 Activity

**Figure 6:**
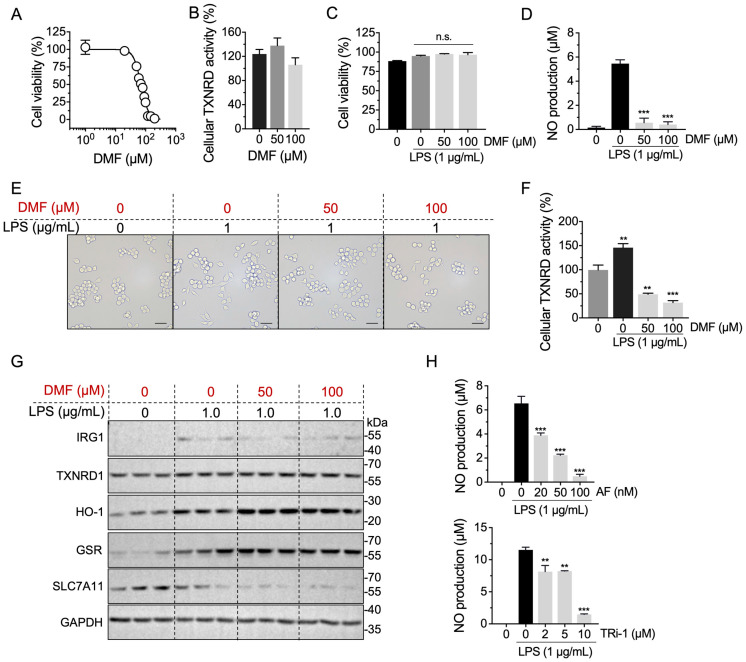
The anti-inflammation effect of DMF is correlated with TXNRD1. (**A**) DMF shows cytotoxicity to RAW 264.7 cells. After DMF treatment for 24 h, MTT assay was used to measure the cell viability of RAW 264.7 cells. (**B**) DMF does not affect cellular TXNRD1 activity in RAW 264.7 cells. Cellular TXNRD activity was determined upon DMF treatment for 6 h. (**C**) DMF presents no significant cytotoxicity to LPS-stimulated RAW 264.7 cells. Cells were treated with 1 μg/mL LPS for 3 h, then the medium was discarded and cells were treated with DMF for additional 24 h, and the cell viability was measured by MTT assay. (**D**) DMF decreases the NO production of RAW 264.7 cells. Cells was treated with LPS for 3 h and then treated with DMF for 24 h in the absence of LPS. (**E**) Cellular morphology image of RAW 264.7 cells upon LPS treatment for 3 h and DMF for additional 3 h; scale bar represents 200 μm. (**F**) DMF inhibits cellular TXNRD activity in combination with LPS treatment. RAW 264.7 cells were stimulated with 1 μg/mL LPS for 3 h, and then cells were treated with indicated concentrations of DMF in the absence of LPS for 3 h. (**G**) Western blot of NRF2-targrt genes. RAW 264.7 cells were stimulated with 1 μg/mL LPS for 3 h, and then cells were treated with the indicated concentrations of DMF in the absence of LPS for 3 h. (**H**) NO production in RAW 264.7 cells upon auranofin or TRi-1 treatment, combined with LPS stimulation. RAW 264.7 cells were stimulated with 1 μg/mL LPS for 3 h and then treated with auranofin (upper panel) or TRi-1 (lower panel) for 24 h in the absence of LPS and the NO production was assayed. **, *p* < 0.01, ***, *p* < 0.001, n.s., not significant.

Previous studies showed that DMF inhibited the inflammation of macrophages by altering NRF2 and NF-κB activity [20,22]. In this study, we found that DMF treatment impaired the cell viability of RAW 264.7 cells (Figure 6A), whereas the cellular TXNRD activity was not strongly affected (Figure 6B). However, upon LPS-stimulation, DMF showed no cytotoxicity towards RAW 264.7 cells (Figure 6C) but could decrease the NO production and alleviate the cellular morphology changes, suggesting an anti-inflammation effect of DMF (Figure 6D,E). Notably, the cellular TXNRD1 activity was decreased (Figure 6F) without observed down-regulation of TXNRD1, indicating the directly inhibitory effect of DMF on TXNRD1 (Figure 6G). What is more, after being treated with LPS, immunoresponsive gene 1 (IRG1), a gene that is highly expressed in mitochondria of macrophages under pro-inflammatory states, was increased and the genes, such as hemeoxygenase-1 (HO-1) and GSR, which are controlled by the KEAP1-NRF2-ARE antioxidant system, were significantly up-regulated, suggesting that DMF activates the NRF2 transcription activity (Figure 6G). Interestingly, the cystine/glutamate transporter SLC7A11 was decreased, which inconsistent with other NRF2 targets. Previous reports showed that DMF induces ferroptosis in cancer cells, which may explain the phenomenon, as SLC7A11 is down-regulated along with the accumulation of lipid peroxidation and ferroptosis process [26,27]. Taken together, these results suggested that DMF directly inhibited cellular TXNRD activity and increased the NRF2 transcription activity.

We then used a potent TXNRD1 inhibitor, auranofin (AF) and TRi-1 to verify the role of TXNRD1 in LPS-stimulated macrophages. The NO production in LPS-induced macrophages was significantly decreased (Figure 6H), indicating that targeting TXNRD1 is a potential means for dousing inflammation.

**Figure 7:**
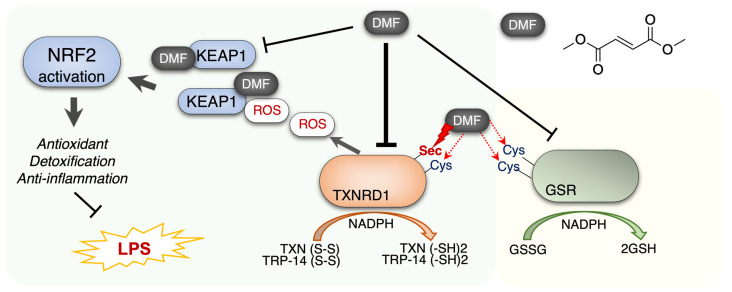
Proposed mechanism of DMF activating the NRF2 transcription but inhibiting TXNRD1 activity. NRF2, Nuclear factor erythroid-2-related factor 2; KEAP1, Kelch-like ECH-associated protein 1; ROS, Reactive oxygen species; TXNRD1, Thioredoxin reductase 1; GSR, Glutathione reductase; LPS, Lipopolysaccharide; DMF, Dimethyl fumarate.

Taken together, our results showed that DMF inhibits TXNRD1’s activity by modifying the Sec and Cys residues in the catalytic motif of TXNRD1. In LPS-stimulated RAW 264.7 cells, the TXNRD1 was up-regulated but DMF directly inhibits the cellular TXNRD1 activity, which may contribute to the anti-inflammatory effect of DMF. We also observed the up-regulation of NRF2 target genes in DMF-treated cells, such as HO-1 and GSR. We speculate that the inhibition of TXNRD1 would promote the NRF2 antioxidant system, which further promotes the anti-inflammation process (Figure 7).

## 3. Discussion

DMF is an electrophilic molecule and a well-known immunomodulatory drug, harboring anti-inflammatory effects via activating NRF2 or inducing electrophile stress through GSH conjugation and Cys-residue modification in enzymes. In the present study, we showed that DMF inhibits the activity of selenoprotein TXNRD1, which further promotes the dissociation of NRF2 from KEAP1, and increases expression of antioxidant enzymes and detoxifying enzymes, such as GSR and HO-1 [28]. As expected, the inhibitory action of DMF on TXNRD1 will enhance its anti-inflammatory effect, precisely as exactly as later we showed in cultured RAW 264.7 cells in vitro.

The discovery of TXNRD inhibitors attracted considerable interest, due to the vital roles of TXNRD1 in tumor progression and metastasis [29,30]. It is quite a big surprise that DMF exhibits an inhibition effect on TXNRD1, given that the profound influences of DMF and such inhibition on cellular function have not been fully understood. Differing from MMF and FA, the action of DMF on TXNRD1 indicates that the electrophilicity of the molecule is vital for TXNRD1 inhibition. Compared with other TXNRD1 inhibitors such as quinones [31,32] or gold-based compounds such as auranofin [33], DMF is not a potent inhibitor of TXNRD1, which also could be inferred by the electrophilicity of DMF. However, it is worth noting that DMF inhibits both the N-terminal and the C-terminal redox active motifs of TXNRD1, distinct from some Sec-targeting electrophilic drugs. Previous works showed that electrophilic molecules modified the Sec^498^ residue of TXNRD1 at the C-terminal producing selenium compromised thioredoxin reductase-derived apoptotic proteins (SecTRAPs) which are devoid of TXNRD1’s activity but can induce rapid cell death by directly generating ROS through its N-terminal NADPH oxidation domain [31,34,35,36,37]. We inferred that the inhibitory effect of DMF on TXNRD1 works more on the cellular function of TXNRD1 rather than directly generating ROS. Meanwhile, the critical role of TXNRD1 implies that activity loss of TXNRD1 will rewire some cellular processes and alter the tolerance of cells to some stress [38,39]. This may be considered means of DMF for inducing cancer cell death and regulating cellular inflammation. 

TXNRD1 and GRX maintain the reduced states of TXN, working together with the GSH system for regulating cell redox balance [40,41,42,43]. We confirmed a major function of TXNRD1 in the current study; it can serve as a negative regulator of the KEAP1-NRF2-ARE antioxidant system as previously reported [44,45]. KEAP1 is a sensor of oxidative stress and electrophile stress [28]. In the canonical pathway, ROS-mediated oxidation or electrophiles-induced modification of the cysteine residue of KEAP1 causes its dissociation from NRF2, which interrupts the ubiquitin-dependent proteasomal degradation and thereby promotes NRF2 activation [46]. However, based on the high chemical reactivity of the Sec residue on TXNRD1, the electrophiles are prone to attack the TXNRD1, and affect the balance of multiple downstream redox signaling and cellular redox activities [40,47]. We propose that the induction of NRF2 by DMF is associated with the inhibition of TXNRD1, which further promotes the anti-inflammation activity of DMF.

## 4. Materials and Methods

### 4.1. Chemicals and Reagents

Auranofin, nicotinamide adenine dinucleotide phosphate (NADPH), fumaric acid (FA), monomethyl fumarate (MMF), and dimethyl fumarate (DMF) were purchased from Yuanye Corp. (Shanghai, China). 5,5′-dithiobis-(2-nitrobenzoic acid) (DTNB), 9,10-phenanthrene quinone (9,10-PQ), and lipopolysaccharide (LPS) were obtained from Sigma-Aldrich (St. Louis, MO, USA). BCA Protein Quantification Kit was purchased from Beyotime Corp. (Shanghai, China). The primary antibodies containing TXNRD1, GSR, xCT were obtained from Proteintech (Wuhan, China) and IRG1 was purchased from Abcam (Cambridge, MA, USA), HO-1 was purchased Cell Signaling Technology (Boston, WA, USA). The secondary antibodies (goat anti-mouse or goat anti-rabbit) were obtained from Bioss (Beijing, China).

### 4.2. Cultured Cell Lines

RAW 264.7 cell line was purchased from Procell Corp. (Wuhan, China) and cultured in DMEM (Procell, Wuhan, China). All the cultured cells were supplemented with 10% FBS, 100 U/mL penicillin, and 100 mg/mL streptomycin in a humidified incubator (Heal Force, Shanghai, China) with an atmosphere of 5% CO_2_ at 37 °C.

### 4.3. Cell Viability Assay

MTT assay was used to measure the cell viability. In brief, RAW 264.7 cells (10,000 per well) were seeded into 96-well plates and incubated at 37 °C overnight. Cells were then incubated with the different concentrations of DMF for the indicated time. Afterwards, the media were discarded, and fresh media containing 0.5 mg/mL MTT were added and continually incubated for an additional 4 h. Finally, the formed formazan crystals were dissolved in 100 μL DMSO and the absorbance was detected using an Infinite 200 PRO plate reader (Tecan, Männedorf, Switzerland) at 570 nm, with 630 nm as a reference.

### 4.4. Expression and Purification of TXNRD1

Human, rat and mouse TXNRD1 were expressed in BL21(DE3) *gor¯* strain with bacterial SECIS element located downstream of the UGA codon. Plasmid pSUABC, which contains *selA*, *selB* and *selC* genes, was co-transformed with the plasmid pET-TRS_TER_ to the strain to improve the Sec insertion efficiency. The recombinant enzyme was induced according to “2.4/24/24” protocol based on the previous report [48,49]. Purification of the enzyme was performed by affinity chromatography using ADP-sepharose followed by a gel filtration using Hiprep 16/60 Sepharcryl S-300. All the enzymes were stored in a TE buffer, pH = 7.5. The specific activities of the human, rat and mouse TXNRD1 are 8.0 U/mg, 22.7 U/mg and 11.0 U/mg, respectively, using DTNB as the substrate. TXNRD1 variants used in this study include Sec^498^ to Cys^498^ (-GCCG, U498C), Cys^497^ to Ser^497^ and Sec^498^ to Cys^498^ (-GSCG, C497S/U498C), Sec^498^ to Ser^498^ (-GCSG, U498S), Truncated form (-GC, △2) and Cys^497^ to Ser^497^ and Sec^498^ to Ser^498^ (-GSSG, C497S/U498S).

### 4.5. Recombinant TXNRD1 Activity Assay

Activity of recombinant rat TXNRD1 was determined by using DTNB reducing assay according to the previous studies [32]. In brief, the reaction mixture contained 2.5 mM DTNB, 300 μM NADPH, and 10 nM W.T. TXNRD1 or 100 nM TXNRD1 mutants as indicated, in 50 mM TE buffer (pH 7.5), and the enzyme activity was calculated by the TNB^−^ formation at 412 nm (εTNB^−^ = 13,600 M^−1^ cm^−1^). In the 9,10-PQ reducing assay, the final mixture contained 30 nM W.T. TXNRD1, 30 μM 9,10-PQ, and 200 μM NADPH in 50 mM TE buffer (pH 7.5). The enzymatic activity of 9,10-PQ reduction was calculated by the NADPH oxidation at 340 nm (εNADPH = 6200 M^−1^ cm^−1^). All reactions were performed in an Infinite 200 PRO plate reader (Tecan, Männedorf, Switzerland) at 25 °C. The same reaction mixture lacking the enzyme was used as a control.

### 4.6. DMF Treatment on Cells

RAW 264.7 cells were seeded into 6-well plates at 400,000 cells per well and treated with different doses of DMF for 24 h. The cells were washed with ice-cold PBS buffer three times and all adherent cells were lysed by the RIPA buffer with 1 mM PMSF protease inhibitor (Solarbio, Beijing, China). Then, the cell lysate was centrifuged at 18,000 rpm at 4 °C for 20 min. Total protein contents were determined by BCA kit (Beyotime, Shanghai, China).

### 4.7. Cellular TXNRD Activity Assay

Cellular TXNRD activity was determined by using end-point insulin assay as previously described [50,51]. In brief, an appropriate amount of cell lysates was added into a master mixture containing 80 mM Hepes buffer (pH 7.5), 15 μM TXN1, 300 μM insulin, 660 μM NADPH, and 3 mM EDTA. A reaction mixture without TXN1 was used as a background control. Samples were incubated at 37 °C for 30 min. Subsequently, 6 M guanidine hydrochloride containing 1 mM DTNB, 10 mM EDTA was added to each well, and an endpoint at absorbance of 412 nm was measured. TXNRD activities of cell lysates were normalized to protein concentration for an accurate comparison.

### 4.8. Glutathione Reductase Activity

Recombinant yeast glutathione reductase (GSR) activity was determined using the oxidized glutathione (GSSG) as the substrate [52]. The final reaction system (200 μL) contained 1 mM GSSG, 2 nM yeast GSR, and 200 μM NADPH. GSR activity was calculated by following the NADPH oxidation at an absorbance of 340 nm (εNADPH = 6200 M^−1^ cm^−1^) using an Infinite 200 PRO plate reader (Tecan, Männedorf, Switzerland) at 25 °C. The same reaction mixture lacking the enzyme was used as a reference.

### 4.9. NO Production Determination

RAW 264.7 cells were seeded into 24-well plates with the density of 100,000 per well and cultured overnight. Next day, cells were incubated with LPS or DMF as indicated. Cell supernatants were collected and added to 96 well plates. 50 μL samples were co-incubated with 50 μL Griess Reagent Ⅰ and 50 μL Griess Reagent Ⅱ (Beyotime, Shanghai, China) for 15 min at room temperature (20 °C ± 1 °C). The amount of NO production was monitored at 540 nm by an Infinite 200 PRO plate reader.

### 4.10. Western Blot Assay

RAW 264.7 cells were lysed with RIPA buffer containing 1 mM PMSF for 20 min, and then centrifugated with 18,000 rpm at 4 °C. The equal mass samples were loaded onto SDS-PAGE gel and then transferred onto the PVDF membranes. The blots were blocked with skimmed milk for 1 h at room temperature and incubated with primary antibodies (TXNRD1 1:6000, GSR 1:3000, IRG1 1:5000, HO-1 1:2000, SLC7A11 1:3000) overnight at 4 °C, then incubated with secondary antibodies (1:7500) for 2 h at room temperature. The ECL solution was used to display the blots by image analyzer (Sagecreation, Beijing, China).

### 4.11. Statistical Analysis

All experiments were performed in triplicate and the data were presented as the mean ± SEM. Statistical differences between the two groups were analyzed by the Stu- dent’s *t*-test. Comparisons among multiple groups were statistically assessed by one-way analysis of variance (ANOVA) and followed by a post hoc Scheffe test. The significant differences between groups were defined as * *p* < 0.05, ** *p* < 0.01, *** *p* < 0.001; n.s. means not significant.

## 5. Conclusions

In summary, our data revealed details of the inhibitory effect of DMF on TXNRD1, which contributes to both NRF2 activation activity and cell death induction activity of DMF. This work also highlighted that TXNRD1 may be a target of some electrophilic immunometabolite derivatives, such as itaconate. The role of TXNRD1 in immunoregulation should be further investigated.

## Data Availability

Not applicable.
